# Engineering *E. coli* cell surface in order to develop a one-step purification method for recombinant proteins

**DOI:** 10.1186/s13568-018-0638-8

**Published:** 2018-06-30

**Authors:** Hamidreza Fasehee, Amin Rostami, Fatemeh Ramezani, Gholamreza Ahmadian

**Affiliations:** 1 Department of Industrial and Environmental Biotechnology, National Institute of Genetic, Engineering and Biotechnology, Shahrak-e Pajoohesh, Pajoohesh Blvd, km 15, Tehran-Karaj Highway, P.O.BOX: 14965/161, Tehran, 1497716316 Iran; 2 Protein Science Group, Gastroenterology and Liver Disease Research Center, Research Institute for Gastroenterology and Liver Diseases, Shahid Beheshti University of Medical Sciences, Tehran, Iran; 3 Physiology Research Center, Faculty of Medicine, Iran University of Medical Sciences, Tehran, Iran

**Keywords:** Surface display, Sortase, Molecular dynamic, Protein purification, *E. coli*

## Abstract

Sortases are enzymes mostly found in Gram-positive bacteria which cleave proteins site-specifically. This feature makes them a promising tool in molecular biology and biotechnology. In this study, using bacterial surface display of recombinant proteins and ability of sortase A in site-specifically cleavage of the amino acid sequences, a novel method for one-step purification of recombinant proteins was developed. Using computational program tools, a chimeric protein containing a metallothionein (mt) and chitin binding domain (ChBD) was attached to the C-terminal domain of the truncated outer membrane protein A (Lpp′-ompA) using sortase recognition site (amino acid residues: LPQTG) as a separator. The structure of the chimeric protein was simulated using molecular dynamics to determine if the LPQTG motif is accessible to the sortase active site. The designed chimeric protein was expressed and purified. The purified chimeric protein was also displayed on the surface of *E. coli* cells. Both purified chimeric protein and the *E. coli* cells displaying Lpp′-ompA-mt-ChBD carrier protein were then treated with sortase to evaluate the efficiency of sortase-mediated cleavage of purified chimeric protein as well as surface displayed-chimeric protein. It is shown that mt-ChBD protein was successfully cleaved and dissociated from Lpp′-ompA carrier and released into the medium after treatment with sortase in both recombinant protein and surface displayed-chimeric protein. The experimental results confirmed the molecular dynamics analysis results. The presented method could be regarded as a novel strategy for one step expression and purification of recombinant proteins.

## Introduction

Bacterial surface display of heterologous proteins has become an attractive strategy for a wide variety of applications like screening of antibody libraries (Cavallari 2017), as a vaccine delivery vehicle (Kalyanasundram et al. 2015), production of cellular adsorbents (Tafakori et al. 2012), and development of cellular biosensors (Liang et al. 2015). In this process, a coding sequence for the passenger protein is fused to the gene encoding a bacterial surface protein. As a result, the passenger protein will be displayed on the surface of the bacteria (Tabuchi et al. 2010). Several surface-anchoring motifs like OmpA, ice nucleation protein (INP) and auto-transporter have the potential of crossing the bacteria membrane and act as the carrier protein, reviewed in (Samuelson et al. 2002).

Among these proteins, OmpA can be found at about 100,000 copies per cell, making it one of the major outer membrane proteins in *E. coli*. OmpA is composed of a globular *C*-terminal domain in the periplasmic space and the eight-stranded anti-parallel β barrel *N*-terminal domain (Vogel and Jahnig 1986; De Mot et al. 1994; Stathopoulos 1996; Pautsch and Schulz 1998) which is exposed to the surface (Wang et al. 2006; Hounsome et al. 2011). OmpA multiple functions have been reviewed elsewhere (Smith et al. 2007), briefly it acts as a virulence factor, plays role in biofilm formation and as a receptor for some bacteriophages. It also plays a structural role in the integrity of the bacterial cell surface (Koebnik et al. 2000).

The modified form of OmpA, called Lpp-OmpA, is a chimeric protein which has been extensively used for surface displaying of different proteins in Gram-negative bacteria (Benhar et al. 2000; Earhart 2000; Irani et al. 2014; Karami et al. 2014; Jeiranikhameneh et al. 2017). This chimeric protein consists of a signal sequence and the first nine *N*-terminal amino acids of the *E. coli* lipoprotein (Lpp) which is fused to a transmembrane domain (amino acids 46–159) of OmpA (Francisco et al. 1992).

In contrast to Gram-negative bacteria, surface proteins in Gram-positive bacteria are anchored to their cell wall. The covalent attachment of proteins to the cell wall is done by Sortase enzymes (Clancy et al. 2010; Hendrickx et al. 2011; Spirig et al. 2011). This family of cysteine transpeptidases cleaves substrate protein at conserved motives. For instance, srtA cleaves its substrate at LPXTG motif, where X represents one of the twenty naturally occurring l-amino acids. The LPXTG motif is followed by a series of hydrophobic residues and a sequence of positively charged residues in the *C*-terminal. Peptide bond cleavage is occurred between the threonine and glycine residues of LPXTG motif. Then the formation of an amide bond is catalyzed by srtA between the carboxyl-group of threonine and the amino-group of the pentaglycine cross-bridge in the cell wall (Proft 2010). As a result, immediately after secretion, the eligible polypeptide will be surface displayed by this anchoring machinery.

In the present study, we designed and developed a one-step system for expressing and displaying the recombinant proteins on the surface of *E. coli* and immediately releasing them from bacterial surface into the medium using the sortase transpeptidase enzyme. We evaluated the feasibility of the process in theory using computational tools followed by the experimental procedure confirming that the recombinant protein can be easily released, collected, and purified from the supernatant of the bacterial culture.

## Materials and methods

### Molecular dynamic analysis

The initial structures of OmpA and metallothionein were isolated from the crystal structure (PDB ID: 1BXW) and NMR structure (PDB ID: 1JJD) respectively. A peptide sequence of LPQTG was added to the extracellular end of OmpA using hyperchem software by binding Leu from LPQTG to the C-terminal of OMPA structure (Froimowitz 1993). Finally, metallothionein and ChBD were added to the LPQTG peptide to construct the chimeric protein Lpp-OmpA-LPQTG-MChBD which is called LO-Mt-Ch hereafter. A 6×His amino acid tag was also added to the C-terminal of the construct. Then the final construct has been simulated by classical simulation of all atoms by GROMACS 5.0.4 software package (Berendsen et al. 1995; Lindahl et al. 2001; Van Der Spoel et al. 2005; Hess et al. 2008; Pronk et al. 2013; Abraham et al. 2015; Páll et al. 2015) and OLPS-AA force field (Jorgensen and Tirado-Rives 1988). The simulations were carried out in a periodic boundary condition (Pratt and Haan 1981) with immersing the molecules in a truncated dodecahedron water box of TIP3P with a margin distance of 9 Å (Jorgensen et al. 1983).

To deal with the long-range electrostatic interactions, the particle mesh Ewald (PME) was adopted during the MD simulations (Kolafa and Perram 1992), and the cut-off distance for non-bonded interactions was set to 9 Å.

The energy minimization was performed in two steps: 1000 cycles of steepest descent and another 1000 cycles of conjugate gradient (Hestenes and Stiefel 1952). The production simulation was performed for 25 ns in the constant temperature and pressure (NPT) with a time step of 2 fs. Temperature and pressure were maintained constant at 300 K and 1 bar by Berendsen thermostat and barostat (Berendsen et al. 1995). Bond constraints were imposed for all hydrogen bonds via SHAKE algorithm (Miyamoto and Kollman 1992). After 25 ns MD simulation, the average structure of the last 2 ns was prepared and the RMSD values of backbone atoms referring to the starting structure were used to monitor the dynamic stability of the MD trajectories. This structure was used as an initial structure for docking. The complex was docked into the binding site of srtA (PDB code of 1t2w was used as an initial structure of srtA) with HADDOCK server followed by MD simulation in the same conditions stated above. Changes of system energy during the simulation confirmed the stability of the system. In order to obtain the interface area between two proteins, the SASA (Solvent Accessible Surface Area) value for every protein was calculated and the SASA value of the complex of the proteins after MD simulation subtracted from it.

### Bacterial strains, plasmids and primers

All primers, plasmids and bacterial strains used in this study are listed in Table 1. Bacteria were cultured in Luria–Bertani (LB) broth or LB agar.

**Table 1:** Primers, plasmids and strains used in this study

Primer, plasmid or strains	Description or Genotype	Source or reference
Primer		
P1 (LPO1-F) NdeI	ggggcatatgaaagctactaaactggtactgggcaacccgtatgttggctttgaaatggg	Tafakori et al. (2012)
P2 (LPO3-R) EcoRI	gggggaattccggtctgcggcagcggaatgccgttgtccggacg	MWG
P1(SRTA3-F) SphI	gggggcatgccaagctaaacctcaaattcc	MWG
P2(SRTA2-R) PstI	ggggctgcagttatttgacttctgtagcta	MWG
Plasmid		
PQE	T5 promoter, 6×His-tag coding sequence, β-lactamase coding sequence, Amp r	Qiagen
PET 26b	T7 promoter, an N-terminal pelB signal sequence for potential periplasmic localization, plus optional C-terminal His·Tag	Novagene
PET 26b-lOAE (pLOAE)	Vector for construction and expressing of chimeric protein containing lpp′-ompA, Elongatus and Chitin Binding domain	Constructed in this study Constructed in this srudy
pSRTA	Vector for construction and expressing of SrtA	NIGEB collection
SrtA∆N	Vector for construction and expressing of SrtAΔ59 (StrAT)	NIGEB collection
pQE30		
Strain		
BL21 DE3	F– ompT gal dcm lon hsdSB (rB- mB-) λ (DE3 [lacI lacUV5-T7 gene 1 ind1 sam7 nin5]	Stratagene
Top 10 *Staphylococcus aureus*	F′[lacIq Tn10 (tetR)] mcrA Δ (mrr-hsdRMS-mcrBC) $\varphi$ 80lacZΔM15 ΔlacX74 deoR nupG recA1 araD139 ∆(araleu) 7697 galU galK rpsL(StrR) endA1 λ−	Invitrogen

### DNA manipulations

Plasmid DNA was isolated from 3 ml of overnight cultures using Roche High Pure Plasmid Isolation Kit (Roche, Switzerland). For larger amount of the plasmid, plasmid DNA was isolated from 50-ml of overnight cultures using the Qiagen Hi-Speed Plasmid Purification Kit (Qiagen, Germany). Sequencing of the plasmid DNA or PCR products was performed at MWG (Germany) (Table 1). All PCR reactions were performed with Fast Start High Fidelity kit (Roche, Germany), according to the manufacturer’s directions, using PTC-150 Mini-Cycler (MJ Research). Following an initial 2 min denaturation step at 95 °C, 35 cycles of 30 s at 95 °C, 30 s at 57 °C, and 1–2 min at 72 °C was performed depending on the length of the target sequence. The amplification was terminated with 7 min incubation at 72 °C. If a PCR fragment was to be sequenced directly without cloning, it was purified with the High Pure PCR Product Purification Kit (Roche, Germany). The PCR products were run on 1% gel (w/v) agarose gels, purified with QIAquick PCR purification kit (Qiagen GMBH, Germany) before cloning. *E. coli* DH5α cells were transformed with ligated plasmids according to CaCl2 method (Sambrook and Russell 2006).

### Construction of recombinant plasmid pLOAEM

The construct pLOAE was made as previously reported with some modifications (Tafakori et al. 2012). Briefly, PCR was used to amplify a DNA fragment containing coding sequence of first nine *N*-terminal amino acids of the *E. coli* lipoprotein fused to coding sequence of amino acids 46–159 from OmpA (primers are listed in Table 1). PCR product was digested with *Nde*I–*Eco*RI restriction enzymes and was ligated into the same sites of plasmid pET26b to make plasmid pLOA. A cassette containing the 5′ *Eco*RI cleavage site, a linker with the sequence of (TTAGCTGAAGCTGCTGCTAAAGAAGCTGCTGCTAAAGAAGCTGCTGCTAAAGCTGCTGCT), the coding region of metallothionein from *Synechococcus elongatus PCC6301*, the region corresponding to the ChBD of Chitinase (ChiS) from *B. pumilus* (a peptide of 40 amino acids from 547 to 586) and 3′ *Xho*I cleavage site was made synthetically (MWG, Germany). This cassette was digested with *Eco*RI–*Xho*I and was cloned into the same sites of recombinant vector pLOA downstream of Lpp′-ompA to establish pLOAE.

A sortase recognition motif (LPQTG) was inserted in a sequence of 3′ primer (PLOA3, listed in Table 1). Lpp′ompA was amplified using PLOA3 and PLOA1 primers. PCR products were digested with *Nde*I–*Eco*RI and inserted into the same sites of pLOAE to establish plasmid pLOAEM (Fig. 2a). The recombinant plasmid was transformed into *E. coli* BL21 (DE3) cells. Following a colony-PCR assay, restriction enzyme analysis was performed on a few recombinant clones and their nucleotide sequence were determined.

### Construction of recombinant plasmid pSrtAT

Primers SRTA3 and SRTA2 were used to amplify the *srtA* gene lacking the first N-terminal 59 amino acid from *S. aureus* subtype *aureus* strain ATCC 25923 using genomic DNA as a template. The DNA fragment was digested with *Sph*I and *Pst*I and cloned into pQE30 (QIAGEN, Germany) to generate psrtA (Fig. 2b). The recombinant vector was then transformed into *E. coli* Top10.

### Purification of the chimeric protein

Overnight cultures grown bacteria (in the presence of 100 μg/ml ampicillin) containing recombinant plasmid psrtA were diluted into 1 l of fresh culture. Protein expression was induced using 1 mM isopropylthiogalactopyranoside (IPTG). Bacterial cells were harvested at OD_600_ = 1.2 by centrifugation at 6000×*g* for 20 min, then washed and suspended in 20 ml of buffer A [50 mM Tris–HCl, 150 mM NaCl (pH 7.5)]. Bacteria were lysed in a French pressure cell at 14,000 lb/in2 and extracts were centrifuged at 32,600×*g* for 15 min. The supernatant was applied to 1 ml of Ni–nitrilotriacetic acid (Ni–NTA) Agarose (Qiagen) that was pre-equilibrated with lysis buffer (50 mM NaH_2_PO_4_, 300 mM NaCl, 10 mM imidazole, 0.05% Tween 20, pH 8.0). After two washes with 10-ml of lysis buffer followed by a single wash with 10-ml of washing buffer (50 mM NaH_2_PO_4_, 300 mM NaCl, 20 mM imidazole, 0.05% Tween 20, pH 8.0), srtA∆N was eluted by 4 ml of elution buffer (50 mM NaH_2_PO_4_, 300 mM NaCl, 250 mM imidazole, 0.05% Tween 20, pH 8.0). Eluted srtA∆N was dialyzed against imidazole in the sortase reaction buffer without NH_2_OH (150 mM NaCl, 5 mM CaCl_2_ and 50 mM Tris–HCl, pH 7.5). LO-Mt-Ch protein with a 6×His tag in its C-terminal was also purified in the same way. SDS-PAGE was used to confirm the purification process. Bradford assay was used for measuring the protein concentration (Bradford 1976).

### SDS-PAGE and western blotting

Sodium dodecyl sulfate–polyacrylamide gel electrophoresis (SDS-PAGE) was performed on a 10% polyacrylamide gel. Proteins were visualized by staining of the gel by Coomassie Brilliant Blue R-250. For western blot analysis, bacterial lysates or purified proteins from different bacterial clones were subjected to SDS-PAGE and transferred to nitrocellulose membrane (Roche, Germany). The membrane was then incubated with primary mouse monoclonal anti-histidine tag-HRP (Roche, Germany) and/or polyclonal anti-chitinase (raised in rabbit). Goat anti-rabbit HRP (Roche, Germany) was used as the secondary antibody conjugate for polyclonal antibody, and the blot was developed with diaminobenzidine tetrahydrochloride (DAB) substrate (Sigma, USA). Equal amount of proteins were used in all experiments.

### Treatment of the purified chimeric protein with sortase

The purified chimeric protein LO-Mt-Ch was incubated in sortase reaction buffer (150 mM NaCl, 5 mM CaCl_2_ and 50 mM Tris–HCl, pH 7.5, 0.2 M NH_2_OH) and srtA was added to start the reaction. The same reaction was carried out without addition of srtA protein as a negative control. Both reactions were incubated at 37 °C for 2 h. SDS-PAGE sample buffer was added to stop the reactions and proteins were separated on SDS-PAGE.

### Treatment of surface displaying recombinant *E. coli* with sortase

To investigate the ability of srtA in cleaving the surface displayed chimeric protein with LPQTG motif, surface displaying recombinant bacteria were treated with sortase. The assay condition was the same as the condition of chimeric protein treatment with sortase, except that the LO-Mt-Ch was displayed on the surface of *E. coli* and its cleavage, as the substrate of srtA, was evaluated.

Overnight cultures of *E. coli* (in the presence of 30 μg/ml kanamycin as a selection marker) containing recombinant plasmid pLOAEM were diluted into 1 l of fresh culture and induced by 1 mM isopropylthiogalactopyranoside (IPTG) at OD_600_ = 0.6. Cells were harvested by centrifugation at 6000×*g* for 20 min, washed, and suspended in 20 ml of sortase reaction buffer in the presence/absence of srtA and were incubated at 37 °C for 2 h. Cells were pelleted at 6000×*g* and the supernatant was evaluated for the presence of cleaved products. SDS-PAGE Sample buffer was added to stop the reactions and proteins were separated on SDS-PAGE. The presence of the cleavage products were confirmed by western blot analysis using polyclonal anti-rabbit antibody raised against ChBD of chitinase protein.

### Cell fractionation

In order to evaluate the changes of the outer membrane protein contents of *E. coli* harboring pLOAEM, cell fractionation was performed. Bacterial cells were harvested and re-suspended in a 25 mM Tris–HCl buffer (pH 8.0) and cell lysates were prepared as follow: lysozyme was added to a final concentration of 50 µg/ml to the cells. Cells were incubated at 4 °C for 1 h, then disrupted by sonication on ice (Cycle = 0.5, power = 75%, 6 × 45″ sonication with 1 min intervals. The crude extract was centrifuged for 10 min at 11,000×*g* to remove cell debris. The membrane and soluble fractions of the cell extract was separated by ultra-centrifugation at 115,000×*g* for 1 h. The supernatant containing the soluble fraction was retained for further outer membrane fractionation. In order to solubilize the inner membrane proteins, the pellet (membrane fraction) was re-suspended in phosphate-buffered saline (PBS) containing 0.01 mM MgCl_2_ and 2% Triton X-100. The suspension was incubated for 30 min at room temperature with gently shaking and the outer membrane fraction was sediment by ultra-centrifugation. Outer membrane fractions were investigated by Coomassie blue stained SDS-PAGE. As a negative control, bacteria were incubated in the same reaction without adding srtA.

### Accession numbers

All sequences were retrieved from NCBI database.

ompA (Accession No. P0A910), ChBD, (Accession No. ABI15082), metallothionein (Accession No. WP_011242577). *S. aureus* subtype *aureus* strain ATCC 25923 (complete genome Accession No. CP00936), sortase A (Accession No. AIO22164),

## Results

**Fig. 1:**
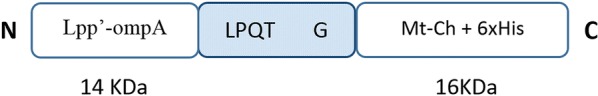
Schematic representation of the chimeric construct LO-Mt-Ch. Carrier lipoprotein Lpp′-ompA is fused to the chimeric protein using srtA recognition site, LPQTG (not drawn to scale). Mt stands for metallothionein from *Synechococcus elongatus* and ChBD represents chitin binding domain of chitinase (ChiS) from *B. pumilus*

The schematic representation of the chimeric protein including selected regions of Lpp′-ompA, metallothionein and ChBD of chitinase (LO-Mt-Ch) is shown in Fig. 1. Lpp′-ompA is fused to the rest of the chimeric protein using the cleavage site for sortase (LPQTG).

### Molecular dynamic simulation

**Fig. 2:**
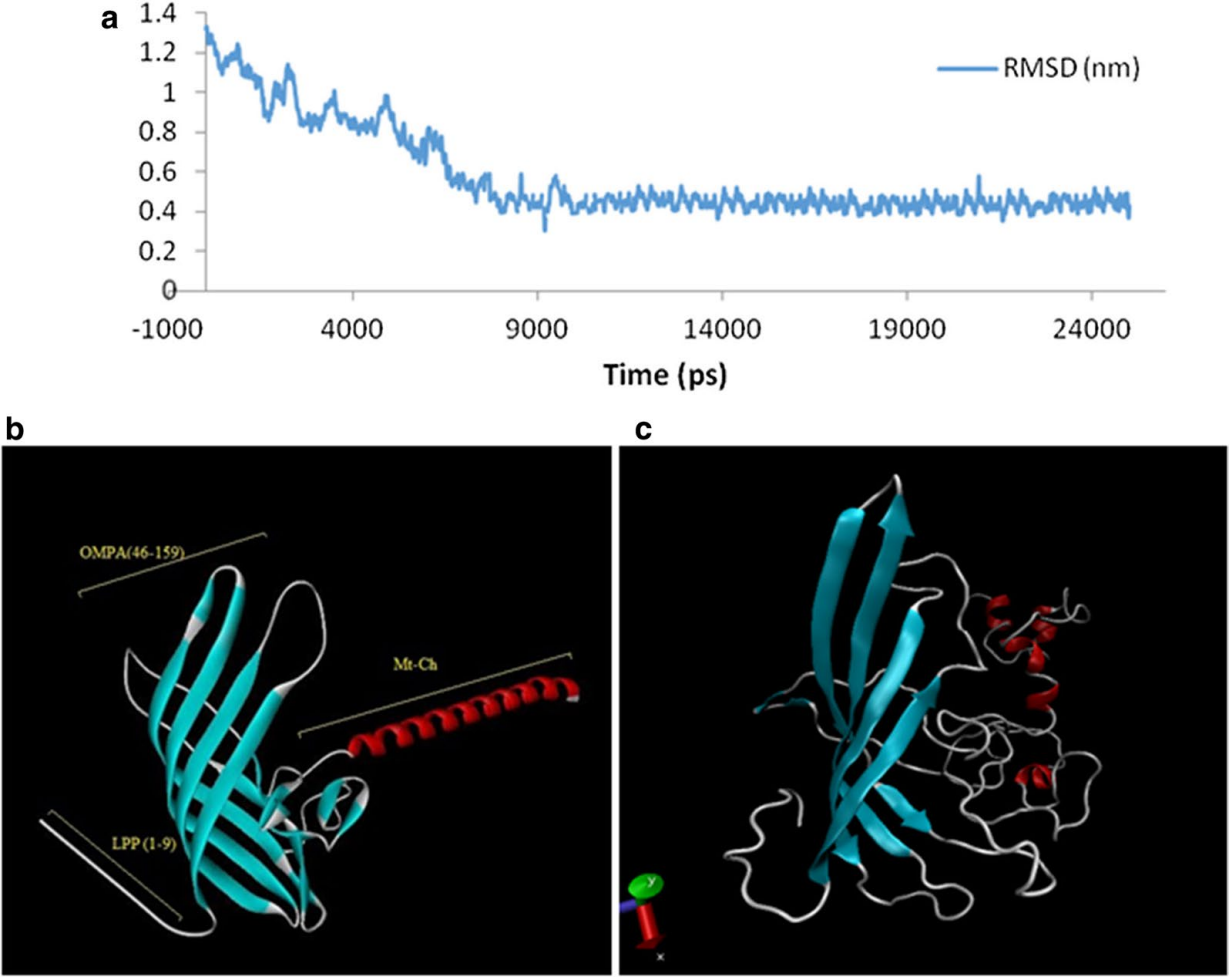
**a** RMSD analysis of complex conformation observed after 25 ns. The complex was stabilized from 10 to 25 ns. **b** LO-Mt-Ch complex before MD simulation that build with hyperchem software and **c** LO-Mt-Ch complex after 25 ns molecular dynamic simulation. Each secondary structure is shown as following color schemes: beta sheets (yellow), alpha helix (blue), 3/10-helix (purple), and turn (white), and bend (green)

The primary structure of LO-Mt-Ch complex was simulated for 25 ns to find the correct secondary structure. Comparing to the starting structure, the RMSD values of the backbone atoms was monitored the stability and convergence of the MD trajectories. Figure 2a shows the RMSD plot for the LO-Mt-Ch complex. The system reached the stable minimum after 10 ns. 3D structure of the LO-Mt-Ch complex is shown before MD simulation in Fig. 2b and after 25 ns MD simulation in Fig. 2c. The result showed that the complex is stable.

**Fig. 3:**
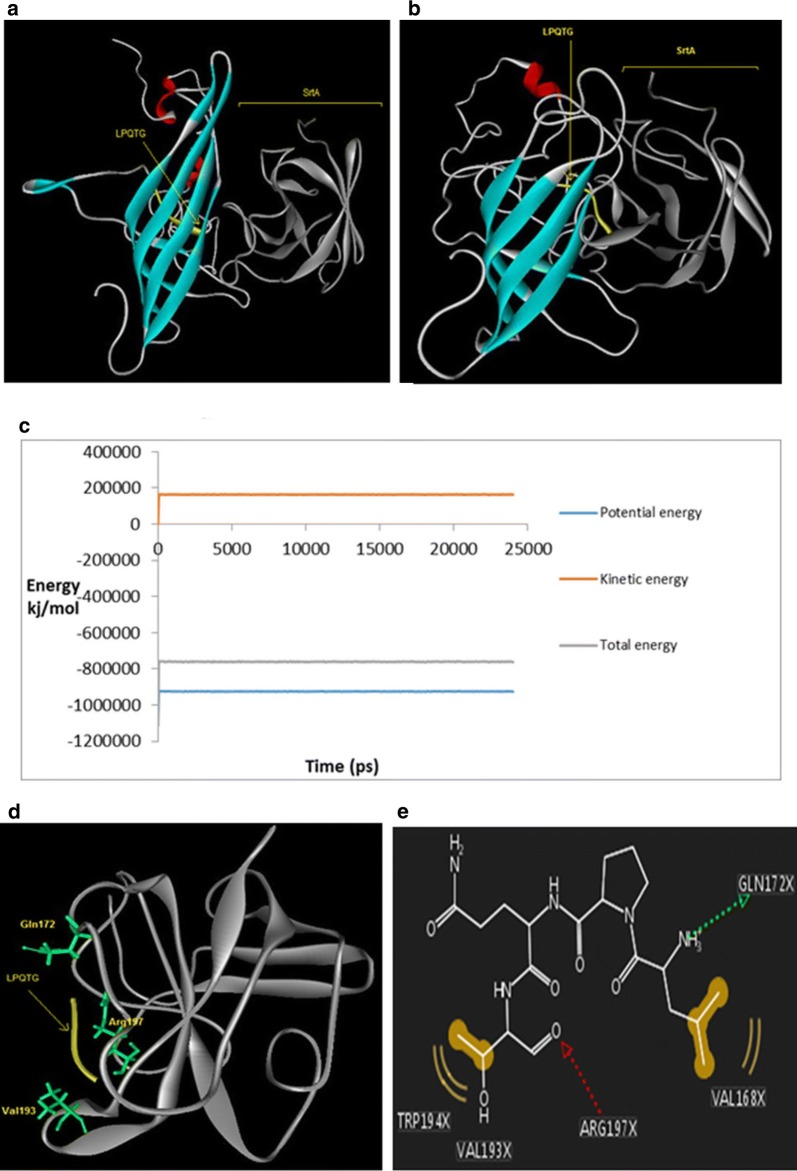
Molecular docking and molecular dynamic simulations for LO-Mt-Ch complex; **a** final structure from docking to the srtA, **b** structure of complex after relaxation showing LPQTG recognized by srtA. **c** Energy changes during simulation. **d** The conformation of residues Gln172, Val193 and Arg197 (green) in binding site during interaction with LPQTG (yellow). **e** 2D interaction diagram for the binding site of srtA with LPQTG. Arg 197 intercts with the bond between Thr and Glys. LO-Mt-Ch complex is gray, srtA is green and LPQTG peptide is yellow with stick atoms

The equilibrated structure of the LO-Mt-Ch complex was docked against srtA binding site with HADDOCK server (Fig. 3) (van Dijk et al. 2012; van Zundert et al. 2016). Although docking analysis can estimate an acceptable binding model, the impact of the solvent and flexibility of the protein are not fully taken into account. The best docked structure with HADDOCK score of − 125.7 ± 3.0 and 117 cluster size was selected for investigating the binding of LPQTG to the srtA binding site by MD simulations. Figure 3 shows the structure of the primary structure (Fig. 3a) and the last frame of MD simulation after 25 ns (Fig. 3b). Energy changes during the MD simulation shows the stability of the system (Fig. 3c). Interactions that occurred between LPQTG and srtA have been elucidated in ribbon form (Fig. 3d). The 2D representation of the average structure of the last 2 ns of simulation indicates that the LPQTG peptide is properly positioned in the srtA active site and interacts with the amino acids of the substrate binding pocket of srtA include GLN172, VAL168, VAL193, TRP194 and ARG197 (Fig. 3e). The SASA value for every protein was 240.617 nm^2^ (155.969 + 84.648) and the SASA value of the complex of the proteins after MD simulation was 211.590 nm^2^. Therefore the interface area is 29.027 nm^2^.

### Treatment of the purified chimeric protein with sortase

**Fig. 4:**
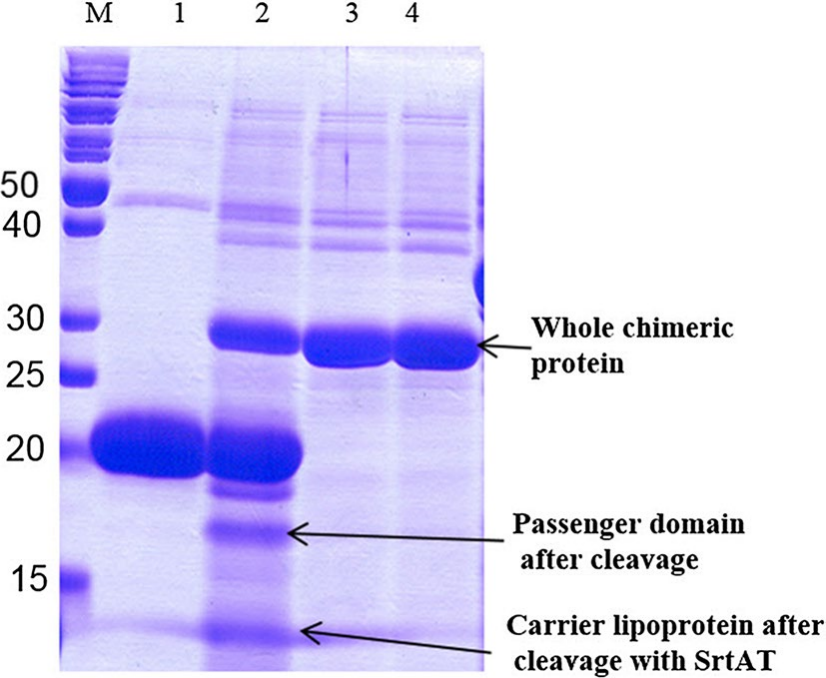
Analysis of sortase activity on LO-Mt-Ch chimeric protein. M: molecular weight marker. Lane 1: recombinant srtA, synthesized in this study. Lane 2: the chimeric protein was partially processed into two fragments of 16 (Mt-Ch) and 14 kDa (Lpp′-ompA) after treatment by sortase (In vitro sortase assay). Lanes 3 and 4: the chimeric protein with out treatment with sortase

SDS-PAGE analysis was used to evaluate the cleavage of LPQTG motif by srtA. As it can be seen in Fig. 4, two separate bands at approximate molecular weights of 14 and 16 kDa are observable which are corresponding to the carrier Lpp′-ompA and passenger domain of the chimeric protein Mt-Ch, respectively. This shows that srtA from *S. aureus* cleaved LPQTG peptides between the threonine and glycine residues and released passenger protein into the medium. A larger band of about 30 kDa corresponds to the uncleaved chimeric protein of LO-Mt-Ch.

### Treatment of surface displaying recombinant bacteria with sortase

**Fig. 5:**
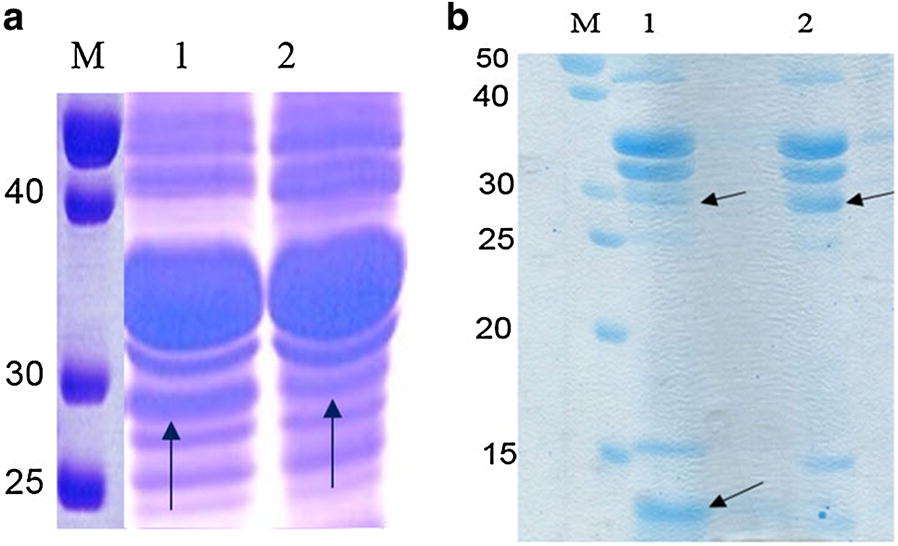
Fractionation of the surface displaying bacterial cells; **a** lane 1: outer membrane fraction of the cell surface from recombinant *E. coli* without sortase treatment. The band corresponding to the whole chimeric protein is indicated with arrow. **a** Lane 2: outer membrane fraction of the cell surface from *E. coli* after treatment with sortase. The density of the band related to the whole chimeric protein is decreased. **b** Lane 1: outer membrane fraction of the cell surface from *E. coli* after treatment with sortase, Lpp-ompA remains in outer membrane fraction (a band of 14 kDa indicated by arrow). Lane 2: outer membrane fraction of the cell surface from *E. coli* without treatment with sortase

The supernatant of bacterial culture medium was investigated for the presence of the chimeric protein (Mt-Ch) which was expected to be released into the medium after treatment of the bacterial cells with srtA. Outer membrane fraction of the pelleted cells was also investigated for the probable changes in content of LO-Mt-Ch and Lpp′-ompA. As it is indicated in Fig. 5, a band of 14 kDa corresponds to the Lpp′-ompA carrier fragment remains in the outer membrane fraction after cleavage of LO-Mt-Ch with srtA.

Results showed that the amount of Mt-Ch in the outer membrane of the cells treated with srtA were decreased comparing to the negative control (Fig. 5). The presence of Lpp′-ompA after treatment of the cells with srtA on outer membrane was also confirmed (Fig. 5).

### Analysis of surface expression and cleavage

**Fig. 6:**
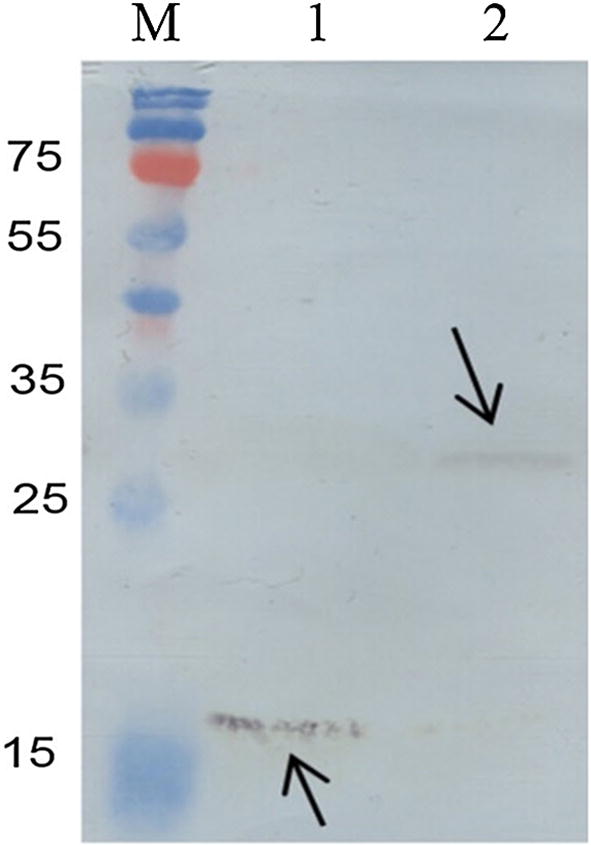
Lane 1, immunoblotting of the supernatant after treatment of the whole surface displayed recombinant bacterial cell with sortase. Lane 2, immunoblotting of the pellet of the recombinant bacteria after treatment with sortase, almost one band corresponding to the complete chimeric protein is observable. This shows that a few amount of the chimeric protein remains on the surface of the bacteria after treatment with sortase

The presence of full-length construct in the supernatant of the surface displaying recombinant bacteria treated with sortase as well as the outer membrane was investigated using western blotting. Polyclonal anti-chitinase antibody against the binding domain of chitinase was used. Observation of a 16-kDa protein band confirms the processing of the construct from LPQTG motif in the presence of srtA (Fig. 6).

Together, these results indicate that the srtA was able to recognize and cleave the surface-anchored LO-Mt-Ch protein bearing the LPQTG motif.

**Fig. 7:**
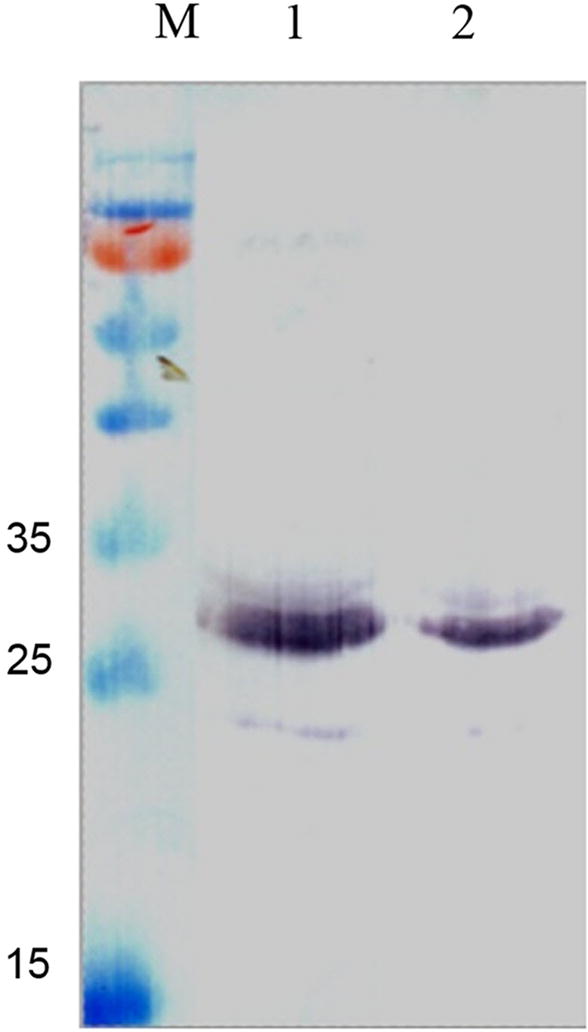
Lane 1, Immunoblotting of the outer membrane fraction without treatment with sortase. Lanes 2, immunoblotting of the outer membrane fraction after treatment with sortase. There is a significant decrease in the density of chimeric protein on the outer membrane

To confirm the decrease observed in the density of LO-Mt-Ch on the outer membrane of the sortase-treated cells, western blotting was carried out using a rabbit polyclonal anti-chitinase antibody against whole ChiS chitinase. As it is shown in Fig. 7, the intensity of the chimeric protein in outer membrane fraction is decreased in the presence of srtA. This confirms the ability of sortase in processing surface anchored LO-Mt-Ch protein and its further dissociation.

## Discussion

Purification of the target protein is a costly and laborious step in large scale recombinant protein preparation in industry. One approach to facilitate the process is by secreting the target protein into the culture medium. Although this strategy makes purification easier, it still requires separation of the protein from the culture medium ingredients and other host secreted proteins.

Bacterial surface display is used for presenting different peptides and proteins on the surface of bacterial cells. Bacterial as well as spore surface display of proteins has attracted interests from biotechnology community for applications in biosensor development (Furst et al. 2017), antibody production (Martineau et al. 1991) and biocatalysts (Tavassoli et al. 2013; Rostami et al. 2017).

Here, we developed a strategy for one step purification of the recombinant proteins based on molecular dynamics prediction and Lpp′-OmpA-mediated *E. coli* surface display in experimental procedure. Among various bacterial displaying systems, the use of OmpA protein motif as signal for protein translocation to the outer membrane of *E. coli* has become an attractive approach. This multifunctional protein is a major component of the *E. coli* outer membrane (Chai and Foulds 1977), and has been successfully used for displaying various proteins on the surface of *E. coli* (Tafakori et al. 2012; Shin et al. 2013; Irani et al. 2014; Qu et al. 2015).

*Escherichia coli* is still the most economical and simplest recombinant expression system used for industrial scale production of recombinant proteins. Previously, we have reported several successful protein displayon the surface of *E. coli* (Tafakori et al. 2012, 2017). In this study we used site-specific cleavage of a surface displayed protein using sortase to facilitate expression and purification of the recombinant protein. Sortases are enzymes mostly found in Gram positive bacteria that can site-specifically cleave LPXTG sequence (LPQTG in this study) and attach proteins to different polymeric surface including bacterial cell wall and also surface-activated polymeric substances (Spirig et al. 2011). It is shown that when peptide LPQTG is placed in the active site of srtA, ARG197 is a critical residue for cleaving the peptide (Zong et al. 2004) which our computational results showed that it was accessible to the srtA active site. Efficiency and specificity of sortase as well as its cost effective usage make it an attractive tool in protein expression industry.

Various strategies have been developed for purification of recombinant proteins (Wingfield 2015). Among them, application of affinity tags for purification of peptides and proteins is a highly popular tool. However, purification of a chimeric protein from highly complex-cell lysate is still a time consuming and costly process. Here, using a combination of the site-specific cleavage activity of sortase enzyme with the bacterial surface display technology, we were able to express a chimeric protein on the surface of the *E. coli* using Lpp′-ompA as a carrier and then specifically cleave the chimeric protein by sortase to release it into the medium (Fig. 6).

Fusion of the chimeric protein to OmpA, one of the major proteins of the *E. coli* surface will ensure the high expression of the target recombinant protein. In the process of sortase cleavage of LPQTG motif, a single Glycine will be added to the protein. However, it is not expected that remaining a single Glycine (after cleavage of LPQTG) at the N-terminal of the protein could change its physicochemical properties.

One main advantage of the recombinant protein expression and purification approach proposed in this study is its simplicity and cost effectiveness as no bacterial lysis step is necessary. In our method, the protein is displayed on the surface of the bacterial cells, released by treatment with transpeptidase enzyme sortase to the environment, and it is simply purified. This approach specially works well for not-secretory proteins, which allows their release into the culture medium instead of their expression inside the cell. Comparing to the densely concentrated whole cell lysate, the medium of the bacterial culture contains just a handful of proteins. Purification of the target protein from such sparse medium may increase the efficiency of purification comparing to its purification from cell lysate.

In summary, docking and molecular dynamics simulation were used to ensure the accessibility of LPQTG peptide (in the structure of LO-Mt-Ch chimeric protein) to the active site of srtA enzyme The chimeric protein was fused to the Lpp′-OmpA using a linker sequence of LPQTG. Then it was shown that treatment of the surface displayed chimeric protein with sortase enzyme can release the chimeric protein into the medium. Using our developed one-step displaying- expression and purification system, the number of stages and the time it takes to express and purify the recombinant proteins are reduced comparing to the conventional methods.

## Abbreviations

Mt: metallothionein
ChBD: chitin binding domain
ompA: outer membrane protein A
Lpp′-ompA: truncated outer membrane protein A
INP: ice nucleation protein
Lpp: lipoprotein
srtA: sortase A
DAB: diaminobenzidine tetrahydrochloride
IPTG: isopropylthiogalactopyranoside
